# A Case Report and Clinical Insights on Encapsulating Peritoneal Sclerosis: A Rare yet Critical Complication of Peritoneal Dialysis

**DOI:** 10.7759/cureus.68344

**Published:** 2024-08-31

**Authors:** M Fawzi Mudarres, Saifatullah Khan

**Affiliations:** 1 Nephrology, University of Iowa Hospitals and Clinics, Iowa City, USA; 2 Nephrology, Hamad Medical Corporation, Doha, QAT

**Keywords:** end stage renal disease (esrd), peritoneal disease, "mass", peritoneal dialysis (pd), renal involvement, encapsulating peritoneal sclerosis

## Abstract

Peritoneal dialysis (PD) offers a valuable alternative to hemodialysis in the management of end-stage renal disease. While PD offers several advantages, such as improved patient autonomy and preservation of residual kidney functions. It has a wide spectrum of complications, which include mechanical ones such as catheter malfunction or migration, hernias and dialysate leak, or infectious complications, which can be limited to exit site and tunnel infections or extend interiorly to cause peritonitis.

One detrimental long-term complication of PD is encapsulating peritoneal sclerosis (EPS), a rare condition characterized by formation of a fibrous cocoon around the bowel loops often initiated by chronic exposure to PD solutions. Other implicated factors include peritonitis, medications and systemic inflammatory conditions. Risk of EPS increases with the duration of PD, particularly after five years. Diagnosis of EPS is challenging and often delayed, given non-specific and wide spectrum of symptoms that may range from loss of appetite to frank signs of abdominal obstruction, which result in significant consequences that can lead to treatment failure and high mortality rate.

Imaging in the form of a CT abdomen is the cornerstone in diagnosis, although many patients are diagnosed intraoperatively during exploratory laparotomy. Treatment is usually directed at eliminating provoking factors and directed therapy based on the disease phase.

In this case, we are discussing a 69-year-old patient presenting with signs of abdominal obstruction and found to have a large cystic lesion compressing small bowels. Eventually, patient obstruction was relieved with draining though interventional radiology after a trial of conservative management failed.

Our goal is to notify our colleagues that we have a high index of suspicion coupled with prompt imaging evaluation that can facilitate early diagnosis, offering hope for improved patient outcomes through timely management strategies.

## Introduction

Peritoneal dialysis (PD) is a well-established modality for renal replacement therapy that has progressively gained traction as a management option for end-stage renal disease. This technique utilizes the peritoneal membrane as a semi-permeable barrier for the elimination of waste products and excess fluid from the body. Given that a patient's peritoneal cavity serves as the dialyzing medium, PD presents a more adaptable and home-based alternative to hemodialysis, fostering enhanced autonomy and quality of life for individuals requiring chronic renal replacement therapy. The primary modalities of PD are continuous ambulatory peritoneal dialysis (CAPD) and automated peritoneal dialysis (APD) [1].

Complications associated with PD encompass both infectious and non-infectious adverse events. Infection, manifesting predominantly as peritonitis, is the most prevalent and critical reported complication, constituting approximately 61% of catheter-related problems [2]. Exit site infections (ESI) and tunnel infections (TI) account for 23%, while catheter obstruction, dislocation, and leakage make up the remainder. The reported overall rate of PD-associated infection ranges from 0.24 to 1.66 episodes per patient-year [2]. Non-infectious complications include those stemming from catheter insertion, such as major bleeding, which occurs in about 2% of cases, particularly in individuals with risk factors like anticoagulation and thrombocytopenia [3]. Lastly, intestinal perforation, a serious but uncommon complication, occurs in 1% or fewer procedures [4].

Encapsulating peritoneal sclerosis (EPS) is a rare but serious long-term complication of chronic PD, characterized by the encasement of bowel loops in fibrous tissue. This condition is associated with significant morbidity and high mortality rates, often leading to ultrafiltration failure and bowel obstruction [5].

The literature presents various clinical scenarios of EPS, illustrating its complexity. For instance, a 71-year-old patient was incidentally diagnosed with EPS during laparotomy for PD catheter removal after peritonitis, primarily exhibiting anorexia and subsequent malnutrition [6]. In another case, a 19-year-old female with a prolonged PD history presented with partial bowel obstruction and was found to have diffuse peritoneal calcifications on imaging, reflecting challenges similar to those in our case [7]. Notably, a retrospective study indicated that 30% of EPS patients succumbed within two months of diagnosis, underscoring the urgency of early detection and intervention [8].

In this context, we elucidate the case of a PD patient encountering bowel obstruction initially suspected to be EPS. However, discontinuation of PD failed to alleviate the obstruction, prompting a computed tomography (CT) scan that revealed a sizable peritoneal cyst characterized by a thickened peritoneal wall exerting pressure on the bowels, necessitating pigtail drainage.

## Case presentation

A 69-year-old male, with a known case of end-stage renal disease secondary to diabetic nephropathy and long-standing hypertension, is currently on maintenance hemodialysis three times per week. He initially began hemodialysis in 2015 through a left brachiocephalic arteriovenous fistula. Based on his preference, he was shifted to peritoneal dialysis in 2016. His course was complicated by recurrent peritonitis, necessitating a return to hemodialysis in 2021. One year later, the patient came for his scheduled hemodialysis session, complaining of several episodes of vomiting the previous day after breakfast and abdominal distension. He also reported constipation for three days.

**Figure 1:**
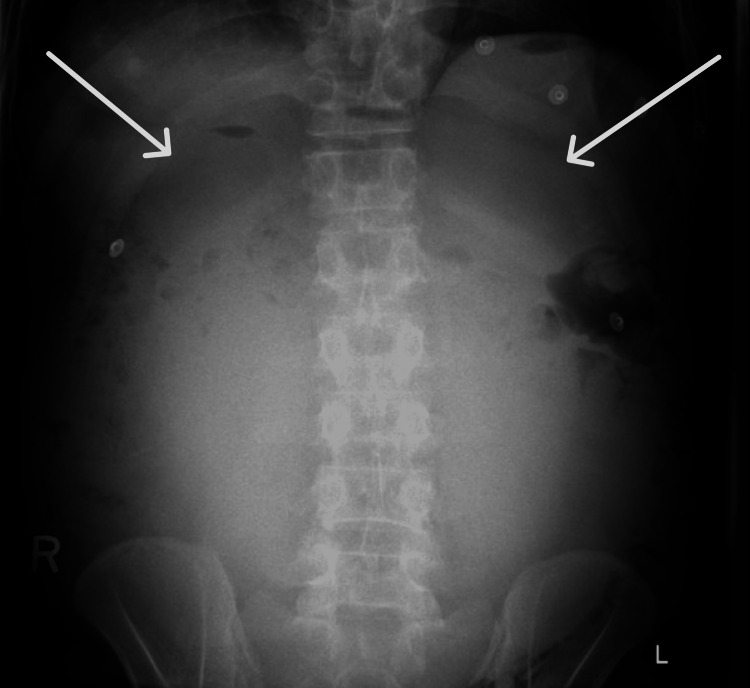
Anteroposterior (AP) abdominal X-ray, erect position White arrows indicate areas of diffuse abdominal opacity, suggestive of a mass

**Figure 2:**
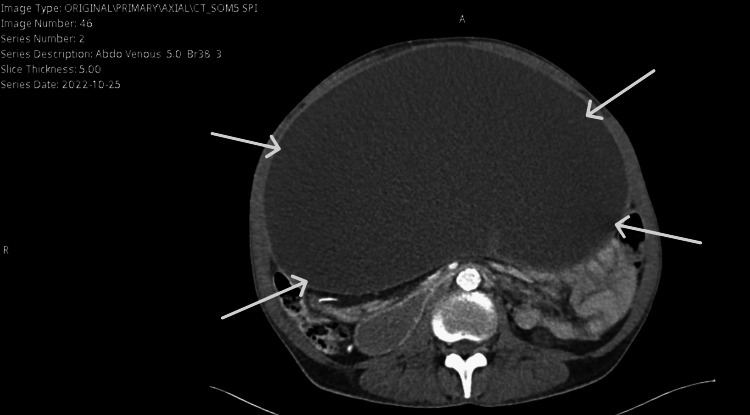
CT Abdomen with contrast, cross-sectional view White arrows point to a large anterior intra-abdominal cystic lesion occupying the entire anterior abdomen.

On examination, the abdomen was markedly distended but soft and lax. Abdominal auscultation revealed exaggerated bowel sounds throughout. Laboratory results, including a complete blood count, showed a normal leukocyte count, hemoglobin of 10.9 g/dL, and normal platelet levels. An abdominal X-ray was performed to rule out intestinal obstruction and showed no air-fluid level (Figure 1). Consequently, a CT scan of the abdomen and pelvis was performed, which revealed a large anterior intra-abdominal cystic lesion occupying the overall anterior abdomen and shifted to the right side. It extended from the L5-S1 level up to the anterior hepatic level with liver contour scalloping, measuring approximately 28.5 x 27 x 16.5 cm in transverse, craniocaudal, and anteroposterior dimensions, respectively, with internal septation (Figure 2). This cystic lesion was significantly compressing all intra-abdominal structures, mainly the bowel loops, with concern for the third part of the duodenum being compressed against the spine posteriorly, causing mild proximal dilatation and delayed gastric contrast emptying. The patient was admitted for a possible intra-abdominal cyst drainage.

**Figure 3:**
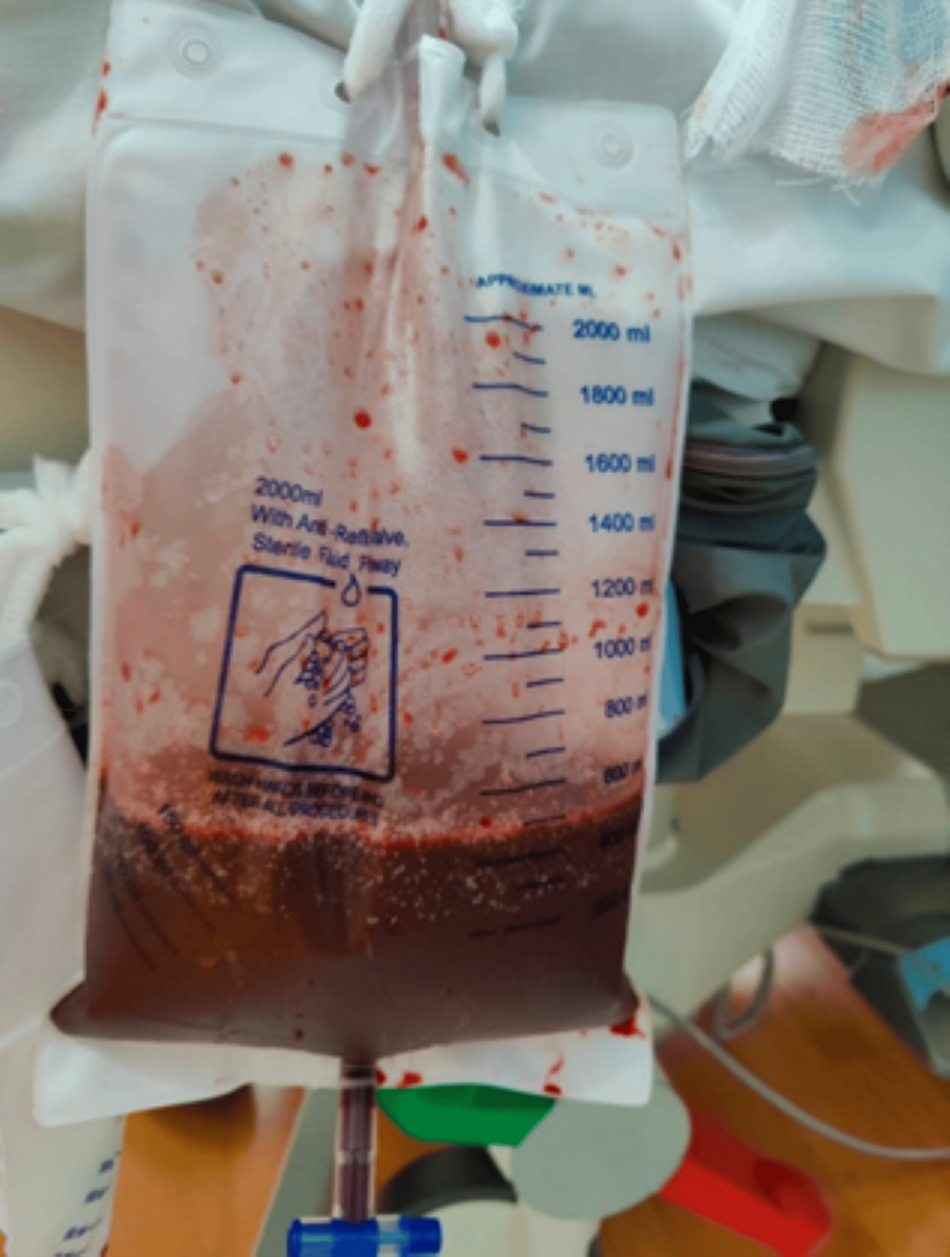
Dark bloody colored fluid had been drained.

Patient was initially treated conservatively, but he developed hypotension on the fifth day, requiring vasopressors and transfer to the Intensive Care Unit. An urgent bedside ultrasound-guided drainage of the cyst was performed to relieve obstruction without complications. Three liters of dark, bloody-colored fluid were drained over 24 hours (Figure 3). The patient felt better after drainage and was able to open his bowels. Two days later, he was stepped down to the medical ward. Microbiology work-up from the body fluid culture was positive for Klebsiella pneumoniae; hence, the patient was given intravenous cefuroxime for seven days, with close observation of the drain output. A total of 14 liters of fluid was drained during this admission. Follow-up imaging with abdominal ultrasound confirmed the resolution of the previously seen encapsulated intra-abdominal cystic lesion, with almost total evacuation through the drainage.

The patient was presumed to have EPS based on a combination of history, imaging, and cystic fluid appearance. He commenced on tamoxifen 10 mg twice daily while resuming the previous hemodialysis regimen without issues. He was subsequently started on prednisolone 30 mg, with a slow tapering over the following six months.

## Discussion

EPS is a rare but severe complication of PD, characterized by progressive inflammation, mesothelial cell loss, submesothelial fibrosis, and peritoneal capillary angiogenesis. Among patients on peritoneal dialysis, the reported incidence varies between 0.7 and 13.6 per 1000 patient-years, with a higher prevalence in males and across all age groups. It is more common in tropical and subtropical countries such as China, India, and Turkey [9,10].

The exact pathogenesis of EPS remains elusive; however, it is postulated to be triggered by exposure to glucose, glucose degradation products, and advanced glycosylation end products in PD fluids [11]. This process results in fibrin deposition on the peritoneum, leading to the formation of a dense capsule that can encase the small bowel, causing obstruction [12]. The primary risk factor for EPS is the duration of PD, with a higher incidence after more than five years, particularly in patients with recurrent severe peritonitis [12,13].

EPS is a gradually progressive and often asymptomatic condition that typically manifests after extended periods of peritoneal dialysis, usually exceeding five years. Recurrent severe peritonitis often exacerbates this disorder, complicating its trajectory. The disease course of EPS is characteristically stepwise, with symptomatology evolving as the condition progresses through its various stages [10,13].

In the early inflammatory phase, patients frequently present with non-specific symptoms such as anorexia, nausea, diarrhea, and intermittent abdominal pain. Physical examinations may reveal blood-tinged dialysate or ascites during peritoneal dialysis exchanges, coupled with progressive volume overload and diminished ultrafiltration capacity. As the disease advances to the late phase, symptoms become more pronounced, with patients experiencing intermittent bowel obstruction manifesting as severe cramping, abdominal pain, constipation, and vomiting. Occasionally, an abdominal mass may be palpable upon examination [14,15].

Laboratory findings in EPS are generally non-specific, although peritoneal dialysis fluid may demonstrate elevated white blood cell counts. Serial peritoneal equilibration tests often indicate a trend toward increased solute transport and reduced ultrafiltration [16]. CT imaging is pivotal in diagnosing EPS, with peritoneal calcification serving as a specific indicator. Additional findings may include bowel thickening, tethering, and dilatation. While a normal CT scan is uncommon in late-stage EPS, it does not preclude the presence of early-stage disease [17]. A presumptive diagnosis is typically based on clinical presentation and characteristic imaging, with definitive confirmation requiring laparotomy and/or laparoscopy [14].

The primary treatment for EPS involves medical management with tamoxifen and prednisone, administered over a course of at least three to four months, followed by a prednisone taper over an additional six to eight weeks [18]. In patients exhibiting severe symptoms, a period of peritoneal rest ranging from four to 12 weeks is often recommended, after which a transition to hemodialysis via a temporary central venous catheter is typically implemented [19]. Some patients may ultimately discontinue peritoneal dialysis altogether. Surgical intervention for acute obstructive complications mirrors the management of bowel obstruction cases. However, chronic symptoms are rarely treated surgically due to the high risk of exacerbating adhesions and unrecognized perforations [20].

The prognosis for EPS is generally poor, with reported mortality rates ranging from 35 to 50%. Nonetheless, transplantation may offer a survival benefit. Supportive care, including nutritional supplementation, along with treatment involving tamoxifen and glucocorticoids, has been associated with improved survival outcomes [15,20].

Despite the absence of reliable preventive measures, potential strategies include minimizing the use of high-glucose dialysate [21]. Given that the major risk factor is the duration of time on peritoneal dialysis-particularly after five or more years-some have advocated for a time limit on peritoneal dialysis. However, this is not recommended in guidelines since the vast majority of patients on peritoneal dialysis do not develop EPS, and the risks associated with hemodialysis may outweigh the potential benefits of preemptively stopping peritoneal dialysis [21]. Studies suggest that patients who may have already started to develop the inflammatory process, which may not yet be clinically evident, could worsen after peritoneal dialysis is discontinued [22]. Some clinicians advocate for resting the peritoneal membrane-stopping peritoneal dialysis and performing hemodialysis for several weeks to months-in patients with risk factors such as repeated episodes of severe peritonitis or decreased ultrafiltration/increased solute transport. However, this practice remains controversial, as EPS may present or worsen after peritoneal dialysis is stopped [22].

In our case study, the patient presented with symptoms of small bowel obstruction, a condition reminiscent of other documented cases in peritoneal dialysis patients. For instance, a 19-year-old female exhibited diffuse calcification on imaging and responded well to conservative management. In contrast, a 26-year-old male faced severe interloop adhesion, which necessitated extensive surgical intervention, including total enterolysis and resection of the diseased terminal ileum [13,23].

Similarly, a 36-year-old male, who had been experiencing abdominal pain for 10 days, required surgical intervention for intestinal obstruction. His preoperative diagnosis of EPS was confirmed both intraoperatively and through histopathological examination [24].

Another noteworthy case involved a 71-year-old male whose primary complaint was anorexia. Initially, CT imaging revealed no abnormalities, but EPS was incidentally discovered during a laparotomy performed to remove a PD catheter following an episode of peritoneal dialysis peritonitis. This case underscores the often subtle and insidious nature of EPS, illustrating the potential for delayed diagnosis. Remarkably, a series of 15 patients found that 13 were diagnosed with EPS incidentally during surgeries for unrelated conditions [8].

These cases highlight the variability in clinical presentation and the critical importance of early imaging in diagnosing EPS. The often non-specific nature of early symptoms and the insidious progression of the disease frequently lead to delayed diagnosis. This highlights the necessity for heightened clinical awareness and timely imaging studies in patients undergoing long-term peritoneal dialysis who present with suspected symptoms of EPS.

## Conclusions

EPS is a complex condition that poses a significant risk to patients undergoing peritoneal dialysis, characterized by high morbidity and mortality rates. The symptoms of EPS can be non-specific, necessitating a high degree of clinical suspicion for timely diagnosis. Despite advancements in understanding its pathophysiology, effective management of EPS remains challenging. Further research is essential to improve the prognosis of this condition, focusing on the development of preventive strategies, refinement of PD techniques, and exploration of innovative treatment modalities.
